# Daily Social Interactions and Well-Being in Older Adults: The Role of Interaction Modality

**DOI:** 10.1093/geroni/igab046.2223

**Published:** 2021-12-17

**Authors:** Gizem Hueluer, Birthe Macdonald, Minxia Luo

**Affiliations:** 1 University of Bonn (Germany); 2 University of Zurich, Zurich, Zurich, Switzerland; 3 University of zurich, Zurich, Zurich, Switzerland

## Abstract

Older adults increasingly use digital communication technologies to stay connected to others. In the present study, we examine the role of social interactions for older adults’ daily well-being focusing on three interaction modalities (face-to-face, telephone, and digital). We use data from 116 participants (age: M = 72 years, SD = 5, range = 65 to 94; 41% women), who reported on their social interactions and well-being over 21 days. Our findings show that frequency of face-to-face interactions is more consistently related to well-being than telephone or digital interactions. On days where participants report more face-to-face social interactions than their own average, they report higher positive affect and lower loneliness than usual. Similar effects are not found for telephone or digital interactions. In summary, our findings suggest that face-to-face social interactions are uniquely relevant to older adults’ daily well-being. We discuss implications of these findings for future research.

